# Neural and Behavioral Evidence for Frequency-Selective Context Effects in Rhythm Processing in Humans

**DOI:** 10.1093/texcom/tgaa037

**Published:** 2020-07-28

**Authors:** Tomas Lenc, Peter E Keller, Manuel Varlet, Sylvie Nozaradan

**Affiliations:** 1 MARCS Institute for Brain, Behaviour, and Development, Western Sydney University, Penrith, Sydney, NSW 2751, Australia; 2 School of Psychology, Western Sydney University, Penrith, Sydney, NSW 2751, Australia; 3 Institute of Neuroscience (IONS), Université Catholique de Louvain (UCL), Brussels 1200, Belgium; 4 International Laboratory for Brain, Music and Sound Research (BRAMS), Montreal QC H3C 3J7, Canada

**Keywords:** beat and meter perception, EEG, frequency-tagging, hysteresis, rhythm processing

## Abstract

When listening to music, people often perceive and move along with a periodic meter. However, the dynamics of mapping between meter perception and the acoustic cues to meter periodicities in the sensory input remain largely unknown. To capture these dynamics, we recorded the electroencephalography while nonmusician and musician participants listened to nonrepeating rhythmic sequences, where acoustic cues to meter frequencies either gradually decreased (from regular to degraded) or increased (from degraded to regular). The results revealed greater neural activity selectively elicited at meter frequencies when the sequence gradually changed from regular to degraded compared with the opposite. Importantly, this effect was unlikely to arise from overall gain, or low-level auditory processing, as revealed by physiological modeling. Moreover, the context effect was more pronounced in nonmusicians, who also demonstrated facilitated sensory-motor synchronization with the meter for sequences that started as regular. In contrast, musicians showed weaker effects of recent context in their neural responses and robust ability to move along with the meter irrespective of stimulus degradation. Together, our results demonstrate that brain activity elicited by rhythm does not only reflect passive tracking of stimulus features, but represents continuous integration of sensory input with recent context.

## Introduction

One of the biggest challenges in understanding brain function is to explain how stable perception is experienced from continuously changing, ambiguous sensory input. To achieve such robustness, it has been proposed that the brain uses prior experience to instantiate expectations, which dynamically interact with the incoming input to shape perception (Dolan et al. 1997; Ahissar and Hochstein 2004; Eger et al. 2007; Esterman and Yantis 2010; Melloni et al. 2011; Holdgraf et al. 2016; de Lange et al. 2018). In particular, stimulus history, that is, recent context, plays a key role in supporting stable perception, especially in the face of degraded sensory input (Snyder et al. 2015). Effects of recent context involve a form of attraction, whereby the perception of the current sensory input is biased towards recently encountered stimuli (Liberman et al. 2016; Cicchini et al. 2018). Such effects have been reported in perception of simple features (Raviv et al. 2012; Fischer and Whitney 2014; Arzounian et al. 2017; Chambers et al. 2017), but also higher-level attributes (Cicchini et al. 2014; Liberman et al. 2014; Suárez-Pinilla et al. 2018; Xia et al. 2018), scene perception (Snyder and Weintraub 2013; Manassi et al. 2017), and reproduction of single temporal intervals (Jazayeri and Shadlen 2010; Cicchini et al. 2012).

Similar robustness to input degradation seems to be present in perception of rhythms (sequences of events in time). When listening to rhythms, particularly in musical contexts, humans often spontaneously organize the incoming sounds in time according to a perceived nested set of periodic pulses, usually referred to as meter (Cohn 2020). Meter perception is considered a cornerstone of temporal prediction and sensory-motor synchronization with rhythm (Toiviainen et al. 2010; Vuust et al. 2018). Traditionally, it has been assumed that whether (and what) metric structure is perceived depends on the acoustic cues in the stimulus, namely distribution of salient acoustic events with respect to the putative pulse positions (Essens and Povel 1985; Parncutt 1994; Toiviainen and Snyder 2003; Tomic and Janata 2008; Large and Snyder 2009). In other words, the more “pulse-like” the physical structure of the sensory input (i.e., the more salient acoustic events are preferentially concentrated at pulse positions), the more likely a meter is perceived. However, recent evidence shows that meter perception is quite robust to input deviations from a pulse-like template (Repp et al. 2008; Sioros et al. 2014; Witek et al. 2014; Câmara and Danielsen 2018), and mapping between the sensory input and perceptual experience not straightforward (London et al. 2017; van der Weij et al. 2017). This indicates that meter constitutes a high-level perceptual phenomenon that shows a degree of flexibility and stability with respect to the physical stimulus.

In line with this view, a growing body of evidence suggests that meter perception is related to fluctuations of neural activity time-locked to the perceived metric pulses (Nozaradan et al. 2011, 2012, 2018; Chemin et al. 2014; Tierney and Kraus 2014; Nozaradan, Mouraux, et al. 2016a; Nozaradan, Peretz, et al. 2016b; Tal et al. 2017; Nozaradan, Keller, et al. 2017a; Nozaradan, Schwartze, et al. 2017b; Lenc et al. 2018; Hickey et al. 2020; Kaneshiro et al. 2020). Importantly, instead of passively tracking the rhythmic structure of the acoustic input, the elicited neural activity is transformed towards selectively tracking the perceived meter, particularly when input deviates from the pulse-like template (Nozaradan, Keller, et al. 2017a). This is manifested as selective enhancement of brain activity elicited at frequencies corresponding to the rates of the perceived metric pulses, relative to activity at other frequencies that are unrelated to the perceived meter but can be nonetheless prominent in the acoustic input (Nozaradan et al. 2011, 2012; Tal et al. 2017). This transformation has been observed already in the human auditory cortex (Nozaradan, Mouraux, et al. 2016a; Nozaradan et al. 2018), and possibly involves functional connections within an extended cortico-subcortico-cortical network (Nozaradan, Schwartze, et al. 2017b). However, how sensory and endogenous signals are continuously weighted to build this neural representation of rhythm remains unknown. The current study addresses this question by directly testing the influence of recent history of auditory stimulation on the selective neural tracking of the perceived meter.

Similarly to other perceptual domains, effects of recent context are arguably at play during meter perception (London 2004). It has been proposed by a number of music theorists that once a stable meter has been established, it tends to withstand ambiguities produced by the continuously changing rhythmic surface of music (Cooper and Meyer 1963; Lerdahl and Jackendoff 1983). While there is evidence suggesting that meter induced by a recent input can affect perception of subsequent time intervals (Desain and Honing 2003; McAuley and Jones 2003), the persistence of meter in the face of a degraded sensory input remains unclear (the general term “degradation” refers here to an input deviation from a template, i.e., how much sensory cues support a particular perceptual interpretation).

In the current study, we tested the impact of recent context on meter processing by creating auditory sequences gradually changing from a regular rhythm (onset structure matching the pulse-like template of a given meter) to a degraded rhythm (irregular onset structure completely ambiguous with respect to the given meter). We also created flipped versions of these sequences, yielding sequences gradually changing from degraded to regular. Electroencephalography (EEG) activity was recorded from participants while listening to these sequences without overt movement. After the EEG session, participants were asked to tap with the hand in time with the perceived pulse of an additional set of sequences constructed with the same algorithm as those used in the EEG session. This behavioral measure therefore indicated the induced metric periodicities across both sets of sequences. Because the envelope spectra of the stimuli were strictly identical across the original and flipped sequences, different EEG spectra across the two sequence directions would provide direct evidence for context-dependent neural representations of rhythm. This context effect would be informative about how the relative contribution of sensory and endogenous signals continuously shapes neural representation of dynamic input, particularly when the sensory information is degraded. We compared groups of musicians and nonmusicians, with the hypothesis that formal musical training would provide the listener with robust ability to perceive meter irrespective of sensory input degradation, thus decreasing sensitivity to recent context (Cicchini et al. 2012).

## Materials and Methods

### Participants

Thirty-two healthy volunteers participated in the study after providing written informed consent. The sample consisted of a group of individuals with no formal musical training (*N* = 16, mean age = 21.1 years, SD = 5.1 years, 9 females), and a group of musically trained participants (*N* = 16, mean age = 24.1 years, SD = 5.4 years, 13 females) with various levels of musical training (mean = 7.2 years, SD = 4.9 years). All participants reported normal hearing and no history of neurological or psychiatric disease. The study was approved by the Research Ethics Committee of Western Sydney University.

### Data and Code Availability

Experimental stimuli and data are publicly available online at https://doi.org/10.6084/m9.figshare.11366120.

### Auditory Stimulation

We created rhythmic patterns by assigning a grid of 12 200-ms events, wherein 8 events were filled with sounds (440 Hz pure tone, 10 ms linear onset and offset ramp) and 4 events with silence in all possible permutations. After removing phase-shifted versions of the same pattern, this resulted in 43 unique patterns. To quantify how well the arrangement of sound events matched a pulse-like metric template, each pattern was analyzed with a model of syncopation proposed by Longuet-Higgins and Lee (1984), as implemented in the synpy package (Song et al. 2015). The syncopation scores were calculated assuming metrical structure comprising nested pulses with rates corresponding to 2, 4, and 12 events respectively (such as in a 3/4 meter). Given these particular pulse rates (i.e., meter frequencies), there were 12 possible ways to align the metric template with each analyzed rhythmic pattern (i.e., 12 meter phases, starting on either of the 12 events constituting the rhythmic patterns). For patterns with highly regular arrangements of sound intervals, the close match of the rhythmic structure and metric template for certain alignments would necessarily result in poor match for other alignments. In contrast, for patterns with highly ambiguous structure, there would be no single alignment resulting in close match between the rhythmic structure and the metric template. Therefore, we used the range of syncopation scores across the 12 possible meter phases (the highest minus the lowest score) as a measure of the regularity of each rhythmic pattern. This value also describes the degree of phase-stability of the meter induced by each pattern. While patterns with large ranges of syncopation strongly encourage perception of particular meter phases over others, there is no such preference for patterns with small syncopation ranges (Povel and Essens 1985; Fitch and Rosenfeld 2007). Based on this analysis, the 43 patterns were then categorized into 8 groups (syncopation ranges {8, 7, 6, 5, 4, 3, 2, 1}, omitting the single rhythm with range of 9), that is, from large syncopation range (regular patterns) to small syncopation range (ambiguous patterns).

Next, we created 57.6-s long sequences, by concatenating 24 patterns randomly chosen (with repetition) from the 43 patterns in such a way that the range of syncopation decreased continuously throughout the sequence. To do so, three different patterns were chosen in each of the eight syncopation range groups from range value 8 to 1. This yielded 3 × 8 = 24 patterns per sequence in total, with gradually decreasing meter phase stability. After randomly choosing a pattern within the desired syncopation-range group, its particular phase was chosen so that the syncopation score continuously increased throughout the sequence, that is, increasing degradation with respect to the meter induced by the patterns (syncopation scores {1, −1, 0, 1, 2, 3, 4, 4} for the eight syncopation range groups). This resulted in a sequence that gradually transformed from regular to degraded without structural changes likely to trigger mental phase-shifts that would markedly reduce the perceived syncopation (e.g., Fitch and Rosenfeld 2007).

**Figure 1:**
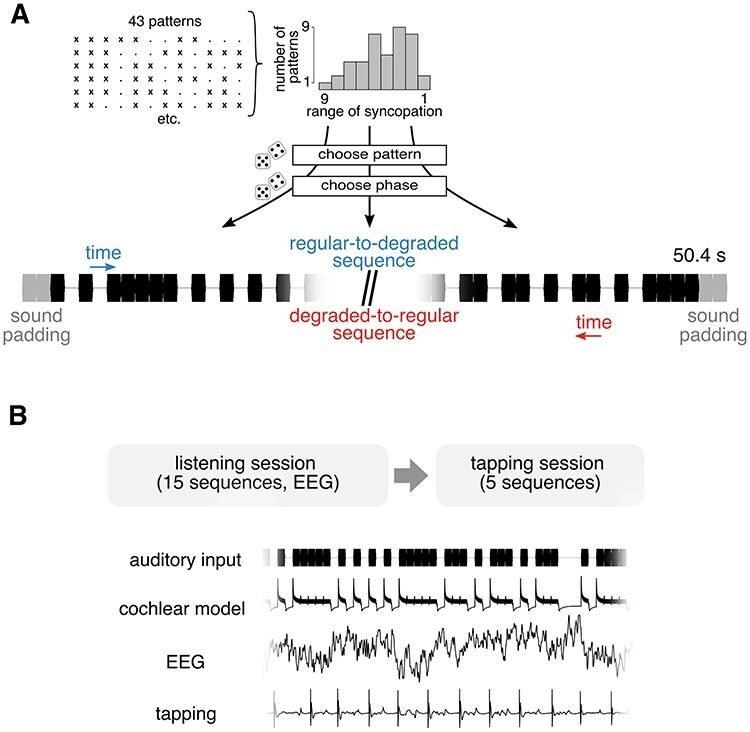
Illustration of the sequence generation method. (*A*) Examples of individual constituent patterns used to construct the sequences. Each pattern contains 8 sounds (depicted as “x”), and 4 silences (depicted as “.”). The patterns were categorized based on the range of syncopation across all 12 possible meter phases (calculated separately for each pattern). Sequences were constructed by randomly sampling patterns according to their range of syncopation. After a pattern was selected, its particular phase (i.e., starting point) was sampled according to the particular syncopation score required. Bottom part of the panel depicts an example of a beginning and an end (padded with 2 sounds) of a single sequence. (*B*) Top panel. Schematic of the experimental design. In the first session, participants listened to 15 sequences and their inverted versions without overt movement, and the EEG was recorded. This was followed by the second session, where participants tapped to 5 additional sequences and their inverted versions. (*B*) Bottom panel. Examples of different signals (in the time domain) analyzed in the current study.

In order to construct sequences with a gradual change in the opposite direction (from degraded to regular), we created a time-inverted version of each 57.6-s sequence, so that the first event became the last event. We also added two sound events at the beginning and end of the sequence, which were excluded from the analyses (see Fig. 1). This prevented spurious differences in the neural response between sequence directions, which could otherwise arise due to increased transient responses to sound events at the beginning of each sequence.

Fifteen unique sequences and their respective inverted versions were generated, forming stimuli for two experimental conditions: the original sequences that evolved from low to high syncopation (regular-to-degraded condition) and their inverted versions that progressed from high to low syncopation (degraded-to-regular condition). Five additional sequences and their inverted versions were constructed for the tapping session. The auditory stimuli were created in Matlab R2016b (The MathWorks) and presented binaurally through insert earphones (ER-2; Etymotic Research) at 75 dB SPL using PsychToolbox, version 3.0.14 (Brainard 1997).

### Stimulus Analysis

#### Syncopation Score

To calculate the evolution of syncopation scores across the generated sequences, the sequences were divided into 14.4-s-long segments (72 events per segment) with 50% overlap, yielding seven distinct segments per sequence. To evaluate whether the corresponding segments in the original and inverted sequences differed in their degree of degradation with respect to the metric template, syncopation scores proposed by Longuet-Higgins and Lee (1984) were calculated for each segment, assuming meter with nested pulses at the rates of 2 and 4 events. This corresponded to the meter used during sequence construction without the slowest pulse, as the individual constituent patterns were not repetitively looped in the sequence. Importantly, syncopation scores are dependent on the particular alignment of the metric template with the analyzed rhythmic pattern (i.e., meter phase). However, the phase of the perceived metric structure was unknown in the current experimental design. Therefore, syncopation scores for each segment were calculated separately after moving the analysis window by −2 to 2 events relative to the first event of the segment (thus including the padding sounds for the first and last segment of each sequence). The minimum syncopation score across the phase shifts was taken, assuming that listeners have a tendency to align their perceptual metric organization in a way that yields the lowest syncopation (Povel and Essens 1985; Fitch and Rosenfeld 2007). Syncopation scores were compared across conditions using a linear mixed model with direction (regular-to-degraded vs. degraded-to-regular) and segment (1–7) as fixed effects. In this test and further statistical tests, for all models including the factor segment as a fixed effect, the order of segments from the degraded-to-regular condition was always reversed in order to compare responses with the exact inverted versions of the same rhythmic stimulus.

The analysis of the syncopation scores calculated for the 15 stimulus sequences used in the EEG session (Supplementary Fig. 1) yielded a significant interaction between the factor direction and segment (*F*_6,182_ = 10.06, *P* < 0.0001, BF_10_ > 100), suggesting that across trials, inversion of the sequences affected only certain segments. Posthoc contrasts revealed that the syncopation score was significantly higher for the degraded-to-regular condition in segment 2 (β = −2.33, *t*_182_ = −4.7, *P* < 0.0001, 95% CI = [−3.31, −1.35]) and 3 (β = −2.67, *t*_182_ = −5.37, *P* < 0.0001, 95% CI = [−3.65, −1.69]), and for the regular-to-degraded condition in segment 4 (β = 1.60, *t*_182_ = 3.22, *P* = 0.01, 95% CI = [0.62, 2.58]). Even though these results suggest that the inversion procedure did not perfectly preserve the theoretically expected amount of syncopation in the sequences, the direction of the effect was opposite to the effect of context we expected to find in the EEG responses. In other words, according to the syncopation scores, there should be slightly better match between the input and metric template in the middle segment in the degraded-to-regular condition.

The procedure used to construct the auditory stimuli in the current study was based on variations in syncopation that assumed a specific metrical interpretation ({2,4} meter with nested pulses at rates of 2 and 4 events). However, there are other possible metrical interpretations of the sequences, which were not considered during stimulus construction. To ensure that the stimulus sequences did indeed change, in theory, from an unambiguous {2,4} meter into highly syncopated sequences instead of converging onto a different meter, we calculated the evolution of syncopation scores across the sequence for two other possible metrical interpretations ({3,6} meter with nested pulses at rates of 3 and 6 events; {2,6} meter with rates of 2 and 6 events). These three different metrical interpretations, ({2,4}, {3,6}, and {2,6}) constitute the simplest nested groupings of the events based on grouping by two or three events. If the sequences modulated into a different meter, then we would expect to find monotonically decreasing syncopation scores for that meter as the sequence progressed from regular to degraded. As shown in Supplementary Figure 2, this was not the case for the two other tested meters, further validating the stimulus construction method that wasused.

#### Cochlear Model

The main motivation for using the exact inversions of the regular-to-degraded sequences to generate the degraded-to-regular sequences was to ensure that the envelope magnitude spectra of the original and inverted sequence were identical (due to the properties of the discrete Fourier transform). This way, differences between the original and inverted sequences in the EEG response across corresponding segments can only be explained by recent stimulus history. To ensure that other nonlinearities in the auditory system (such as adaptation) were not likely to explain the differences between the original and inverted sequences in the EEG response, the stimuli were analyzed with a cochlear model. The model consisted of a Patterson-Holdsworth ERB filter bank with 100 channels (Patterson and Holdsworth 1996), followed by Meddis’ hair-cell model (Meddis 1986), as implemented in the Auditory Toolbox for Matlab (Slaney 1998). The output of this model is designed to approximate sound representation in the auditory nerve, after narrowband filtering at the level of cochlea and nonlinearities introduced at the hair-cell level (adaptation, compression). The output of the cochlear model for each trial and sequence direction was segmented into seven 14.4-s-long segments with 50% overlap (as for calculation of the syncopation scores). The obtained time-domain signals were averaged across trials separately for each 14.4-s segment and sequence direction, and transformed into the frequency-domain using fast Fourier transform (FFT, yielding a spectral resolution of 1/14.4 s, i.e., ~0.069 Hz). The resulting magnitude spectra were then averaged across cochlear channels.

**Figure 2:**
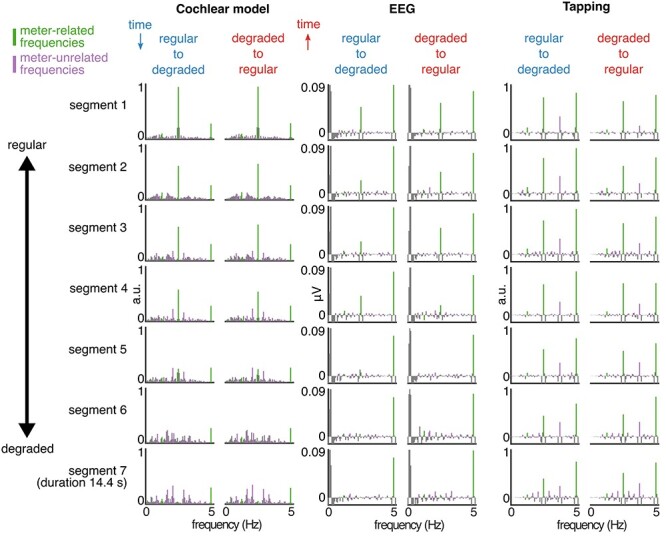
Cochlear model, EEG, and tapping spectra. The data are averaged across trials and plotted separately for each segment and sequence direction. The segments from the degraded-to-regular condition are displayed in reverse order to facilitate comparison across conditions (this way the segments with the same stimulus envelope spectra are aligned). The cochlear model output (Left) shows highly similar spectra across sequence directions, with decreasing prominence of meter-related frequencies (green) and increasing prominence of meter-unrelated frequencies (purple) as the sequence changes from regular to degraded. The EEG response (Middle) averaged across all channels and participants contains peaks at the frequencies present in the cochlear model output, with decreasing prominence of meter-related frequencies in the degraded segments. The tapping response (Right) averaged across participants shows prominent peaks at meter-related frequencies even in the degraded segments.

As depicted in Figure 2, none of the obtained spectra showed clear peaks emerging from the spectral background, except at the frequency of individual events (5 Hz), and half this rate (2.5 Hz). This was due to the fact that none of the patterns making up the sequences were consistently repeated within the sequence, thus yielding no prominent periodicities in the sequences except those related to individual events and successions of two events. As the sequences gradually transformed from regular to degraded, the prominence of the peak at 2.5 Hz decreased over the segments, and the spectral energy spread across other frequencies, thus indicating, as intended, the absence of prominent cues to any particular higher-order structure beyond the eventrate.

To make sure that the output of the cochlear model was not significantly different between sequence directions, especially at the frequencies related to the induced meter, we measured the amplitude at specific frequencies in the obtained spectra. These frequencies corresponded to different possible groupings of the events comprising the sequence, that is, considering cycles of 12 events (0.416 Hz) and 16 events (0.312 Hz) and their harmonics up to 5 Hz (individual event frequency). From this set (*N* = 21 frequencies), a subset of frequencies was categorized as related to the induced meter (1.25, 2.5, and 5 Hz, as confirmed by the tapping session; see section Tapping Analysis). These meter-related frequencies represent nested grouping of the individual event rate (5 Hz) by 2 (2.5 Hz) and 2 (1.25 Hz), thus corresponding to the meter used to construct the sequences (as for the syncopation score calculation). All other frequencies were considered meter-unrelated. The amplitude at each frequency was extracted either at the exact frequency, if a bin was centered at that frequency (14 frequencies), or otherwise as a maximum value from the two closest bins. The 21 extracted amplitudes were *z*-scored as follows: (*x* − mean across the 21 frequencies)/SD across the 21 frequencies. This standardization evaluated the magnitude at each frequency relative to the other frequencies, and therefore allowed us to quantify how much a particular subset of frequencies (here meter-related frequencies) stood out relative to the whole set of frequencies. Because this measure is invariant to differences in unit and scale, it also enabled us to objectively measure the relative distance between stimulus representation at the earliest stages of the auditory pathway (estimated with the cochlear model) and the elicited EEG response.

The relative prominence of meter-related frequencies in the cochlear model output (considering the whole set of 21 extracted frequencies) was calculated as a mean *z* score at 1.25, 2.5, and 5 Hz. These meter-related *z* scores were compared between the two sequence directions across segments to ensure that the inversion of the stimulus was unlikely to introduce significant differences in the prominence of meter frequencies at the earliest stages of the auditory pathway. For this comparison, the *z*-scored amplitudes were extracted in the way described above but separately for each trial (i.e., without first averaging across trials in the time domain), and fitted with a mixed model (fixed effects direction and segment). There were no significant differences between the original and inverted condition (main effect of direction, *F*_1,182_ = 0.01, *P* = 0.92, BF_10_ = 0.15; interaction of direction and segment, *F*_6,182_ = 0.64, *P* = 0.7, BF_10_ = 0.07). This result suggests that nonlinearities at the early stages of the auditory pathway are unlikely to account for any effects of context in the EEG responses.

The same analyses performed on the five sequences used in the tapping session suggested similar differences in syncopation scores, including higher syncopation score for degraded-to-regular condition in segment 2 (β = −2.8, *t*_52_ = −2.96, *P* = 0.03, 95% CI = [−4.7, −0.9]) and 3 (β = −3, *t*_52_ = −3.17, *P* = 0.02, 95% CI = [−4.9, −1.1]), and no significant effects involving the factor direction for the analysis with cochlear model (*P*s > 0.82, BFs_10_ < 0.25).

### Experimental Design and Procedure

The experiment consisted of an EEG and a tapping session directly following each other. In the EEG session, participants were presented with the 15 sequences and their inverted versions in random order with regular-to-degraded and degraded-to-regular trials alternating (counterbalanced across participants). Participants were seated in a comfortable chair with their head resting on a support, and asked to avoid any unnecessary movement. The support made contact with the head just below the most inferiorly positioned electrodes in order to prevent artifacts in the recorded EEG signals. Participants were asked to focus on the regular pulse in the auditory stimuli, and after each trial, to rate (on a scale from 1 to 5) how difficult on average they thought it would be to tap along the pulse in that trial. To further encourage attention to the temporal properties of the stimuli, participants were also asked to detect slight transient decrease of tempo randomly inserted in two additional trials that were not included in the analyses. Before the EEG session, the experimenter provided examples of pulse in popular music and artificially constructed rhythms, to make sure participants understood thetask.

After the EEG session, participants were presented with five additional sequences and the respective inverted versions (as for the EEG session, with random order, sequence direction alternating, counterbalanced across participants), and were asked to tap the regular pulse they perceived in the sequences using the index finger of the preferred hand. Participants were instructed to tap any pulse they perceived in the rhythmic sequence, as long as the pulse they tapped was (1) isochronous and (2) synchronized to the stimulus sequence. They were allowed to start and stop tapping within a trial depending on whether they perceived a periodic pulse or not, and change the period or phase of the pulse at any point. Tapping was performed on a custom-built response box containing a piezoelectric sensor that converted the mechanical vibrations of the box due to the impact of the finger into electrical signals, which were recorded as audio files.

### E‌EG Recording and Preprocessing

The EEG was recorded using a Biosemi Active-Two system (Biosemi) with 64 Ag-AgCl electrodes placed on the scalp according to the international 10/20 system, and two additional electrodes attached to the mastoids. Head movements were monitored using an accelerometer with two axes (front-back and left–right) attached to the EEG cap and recorded as two additional channels. The signals were digitized at a 2048-Hz sampling rate and downsampled to 512 Hz offline.

The continuous EEG signals were high-pass filtered at 0.1 Hz (fourth order Butterworth filter) to remove slow drifts from the signal. Independent component analysis (Bell and Sejnowski 1995; Jung et al. 2000) was used to identify and remove artifacts related to eye blinks and horizontal eye movements based on visual inspection of their typical waveform shape and topographic distribution (two components removed for 14 participants, one component for 18 participants). Channels containing excessive artifacts or noise were linearly interpolated using the three closest channels (one channel interpolated for two participants, four channels for 1 participant). The cleaned EEG data were segmented into 57.6-s long epochs, starting from 0.4 s relative to trial onset (i.e., discarding the two padding sound events, see above section Auditory Stimulation and Fig. 1). If an epoch contained excessive artifacts it was discarded from further analyses (1 epoch for 1 participant), as well as the epoch for the trial with inverted version of the corresponding stimulus sequence. The epochs were then further segmented into seven 14.4-s long segments with 50% overlap (as for the auditory stimulus analysis), rereferenced to the common average, and averaged across trials in the time domain separately for each sequence direction, segment, and participant. Time-domain averaging was performed to increase the signal-to-noise ratio of the neural response by canceling signals that were not time-locked to the stimulus (Mouraux et al. 2011; Nozaradan et al. 2011, 2012). The EEG preprocessing was carried out using Letswave6 (www.letswave.org) and Matlab.

### Frequency-Domain Analysis of EEG Response

For each participant, sequence direction, and segment, the EEG signals were transformed into the frequency domain using FFT. The obtained EEG spectra can be assumed to consist of a superposition of (1) responses to the stimulus concentrated into narrow peaks and (2) residual background noise smoothly spread across the entire frequency range. To obtain valid estimates of the responses, the contribution of noise was minimized by subtracting, at each frequency bin, the average amplitude in the second neighboring bin either side of it (Mouraux et al. 2011; Xu et al. 2017).

Because the meter-unrelated frequencies did not form prominent narrow peaks in the output of the cochlear model, it was important to ensure that the noise subtraction would not selectively suppress meter-unrelated frequencies in the EEG spectra (which could lead to spurious increase in the relative prominence of meter frequencies if there was an overall increase in response gain). A control analysis conducted on the EEG spectra obtained without noise subtraction yielded similar results to the analysis incorporating noise subtraction (see Supplementary Results), showing that this processing step alone could not explain our results. The noise-subtracted spectra were averaged across all channels to avoid electrode-selection bias and to account for individual differences in response topography.

To assess the relative prominence of the specific frequencies in the EEG responses elicited by the auditory stimuli, amplitudes at the 21 frequencies corresponding to different possible metric interpretations were then extracted from the spectra and *z*-scored in the same way as for the auditory stimulus analysis. A higher *z* score at a specific frequency indicates more prominent amplitude at that frequency relative to the whole set of 21 frequencies in the EEG response. Mean *z*-scored amplitude at frequencies related to the induced meter (5, 2.5, and 1.25 Hz, as theoretically expected based on the sequence generation algorithm and as indicated by tapping analysis) was taken as a relative measure of selective neural tracking of the meter periodicities (control analysis with raw EEG amplitudes yielded similar results to the analysis with *z* scores, see Supplementary Results). The mean meter-related *z*-scored amplitudes were compared across sequence directions and segments, by fitting a mixed model (fixed effects direction, segment, and musical training). We expected to find a decrease in the prominence of meter-related frequencies in the segments with higher degradation, as in the auditory stimulus. Importantly, we used additional posthoc contrasts to test whether the EEG response was affected by the direction of the sequence, by comparing the prominence of meter frequencies in segment one (most regular rhythm) with all subsequent segments, separately for each sequence direction. We hypothesized that in the regular-to-degraded condition, the decrease would take place in segments with higher amounts of degradation compared with the degraded-to-regular condition. We also directly compared segments that had identical sound envelope spectra across sequence directions, to assess whether the EEG response at meter-related frequencies would be enhanced for particular segments in the regular-to-degraded condition.

To further show that cochlear processing was unlikely to explain the effect of context in the EEG responses, the two signals were directly compared after standardization (*z*-scoring). In order to use the same processing pipeline for the EEG and cochlear model (see section Stimulus Analysis), the cochlear model spectra were noise-subtracted (second bin on each side) before *z*-scoring the magnitudes across the meter-related and meter-unrelated frequencies. Subsequently, the difference in meter-related *z* scores between the cochlear model and the EEG response was calculated separately for each sequence direction, segment, and participant. The difference scores were compared between sequence directions, segments, and levels of musical training with a mixed model, and posthoc contrasts compared the difference score between directions separately for each segment. Hence, if the EEG responses were fully explained by cochlear processing, the obtained scores should not significantly differ between the two sequence directions.

### Tapping Analysis

Tap times were extracted by locating points in the continuous signal from the tapping sensor, where the (1) amplitude was increasing, (2) amplitude exceeded a threshold set manually for each participant, and (3) the amount of time from the previous detected point was larger than a constant set manually for each participant. These points corresponded to the tap onsets, that is, the times where the finger hit the responsebox.

To quantitatively evaluate the meter periodicities participants synchronized to, the median intertap interval (ITI) was calculated separately for each sequence direction and participant. The value was then compared with three possible meters each consisting of three nested periodicities (nested pulses at rates of {2,4}, {2,6}, and {3,6} events, corresponding to periods {200, 400, 800}; {200, 400, 1200}; and {200, 600, 1200} ms, respectively) by taking the minimum percent difference between the median ITI and the three possible periodicities comprising each meter. This minimum difference score was compared across meters and sequence directions using a mixed model. The meter that yielded the smallest difference score was considered to be the meter predominantly induced by the stimulus construction method.

To assess how well participants synchronized to the meter periodicities, it was important to consider the challenges stemming from the nature of the tapping task, whereby participants were free to tap any periodic pulse they perceived and could start and stop tapping at different points within a trial. Therefore there was no a priori information about the particular period and phase they were tapping, and the number of executed taps could differ between trials. Additionally, the tapped period and phase could change between and within individual analysis windows, without necessarily implying poor synchronization to the meter.

To provide a measure of synchronization insensitive to infrequent changes in tapping phase within the analysis windows, an ITI-error index was calculated separately for each participant, sequence direction, segment, and trial. This was done by first removing ITIs longer than 2 s and finding the minimum percent difference between the median ITI and the three periodicities within the predominantly induced meter (i.e., 200, 400, 800 ms, see Results section). The period closest to the median ITI was considered the pulse chosen by the participant for the analyzed window, and ITI-error was calculated as percent difference between this period and each individual ITI. The ITI-errors were averaged across trials and analyzed using a mixed model with direction, segment, and musical training as fixed effects. If the participant tapped with a fixed period corresponding to one of the metric pulses, but changed the alignment of this pulse with respect to the rhythmic stimulus at some point in the analysis window, ITI-error would remain low. Hence, the main advantage of this measure was its robustness to changes in tapping phase. However, if the participant changed the tapping period within the analysis window to another metric pulse, the ITI-error would becomehigh.

Thus, in order to account for this, the tapping was also analyzed in the frequency domain. This evaluated synchronization at meter-related frequencies at the level of behavioral output with a method directly comparable with the auditory stimuli and EEG responses. The main advantage this frequency-domain analysis was its robustness to changes in tapping period within the analysis window, as tapping either metrical pulse would result in energy distributed solely across meter-related frequencies. However, the method was sensitive to phase changes, as changes in tapping phase within the analysis window would lead to decreased Fourier magnitude at the tapping frequency. This is in contrast with ITI-error, which was robust to phase changes but sensitive to changes in tapping period. Moreover, continuous signals from the tapping box contained information about tapping intensity (amount of accentuation of each tap), thus potentially revealing periodicities in the behavioral response that would remain hidden when analyzing ITIs. Continuous signals from the response box recorded during the tapping session were segmented the same way as the EEG signals, averaged across trials in the time domain, and transformed into the frequency-domain using FFT. The contribution of background noise was minimized, as for the EEG, by subtracting the average magnitude in the second neighboring bin either side of each frequency-bin. The resulting magnitude spectra were averaged across trials, and magnitudes at meter-related and meter-unrelated frequencies were extracted and *z*-scored as for the EEG analysis. Mean *z*-scored amplitudes at meter-related frequencies were compared across segments, sequence directions, and levels of musical training, by fitting a mixed model. The persistence of the tapping synchronization across different amounts of syncopation was assessed using posthoc contrasts that compared the prominence of meter-related frequencies in the first segment with all subsequent segments. To further understand the evolution of the tapping response over segments, the prominence of meter frequencies was also compared across all pairs of successive segments.

### Head Movement Analysis

To evaluate the extent to which unintentional head movement artifacts could explain the observed EEG results, the data from the accelerometer were segmented the same way as EEG signals and transformed into the frequency-domain separately for each movement axis. The resulting spectra were averaged across the two axes, and mean magnitudes at meter-related frequencies were extracted and further analyzed as for the EEG responses. This control analysis confirmed that the observed EEG effects were unlikely to be explained by head movement artifacts (see Supplementary Results).

### Statistical Analyses

The statistical analyses were performed using linear mixed models with lme4 package in R (Bates et al. 2015). Each participant was included as a random-effect intercept (in case of stimulus analyses, the intercept was modeled as a random variable across trials). For models including the factor segment as a fixed effect, the order of segments from the degraded-to-regular condition was always reversed in order to compare responses with the inverted version of the same acoustic stimulus. Posthoc multiple comparisons were computed using emmeans package (Lenth 2018). The Kenward–Roger approach was used to approximate degrees of freedom and Bonferroni correction was used to adjust for multiple comparisons. Complementary to the null-hypothesis significance tests with mixed models, we also calculated Bayes factors to quantify the evidence in favor of the alternative hypothesis over the null hypothesis (BF_10_), as implemented in the package BayesFactor for R (Morey and Rouder 2014).

## Results

### Tapping

#### Median ITI Analysis

The tapping task confirmed theoretical expectations about the meter periodicities induced by the auditory stimulus sequences. The difference between the median ITI and possible meter periodicities varied significantly across the different possible meters (*F*_2,155_ = 19.65, *P* < 0.0001, BF_10_ > 100). Posthoc comparisons showed that the median ITI was significantly closer to the {2,2} meter than the {3,6} meter (β = −13.22, *t*_157_ = −5.39, *P* < 0.0001, 95% CI = [−19.15, −7.28]) and {2,6} meter (β = −13.54, *t*_157_ = −5.52, *P* < 0.0001, 95% CI = [−19.47, −7.61]). These results further justify the selection of meter-related frequencies (5 Hz, 5 Hz/2 and 5 Hz/4, corresponding to the rates of one, two, and four individual events respectively) for the frequency-domain analyses.

**Figure 3:**
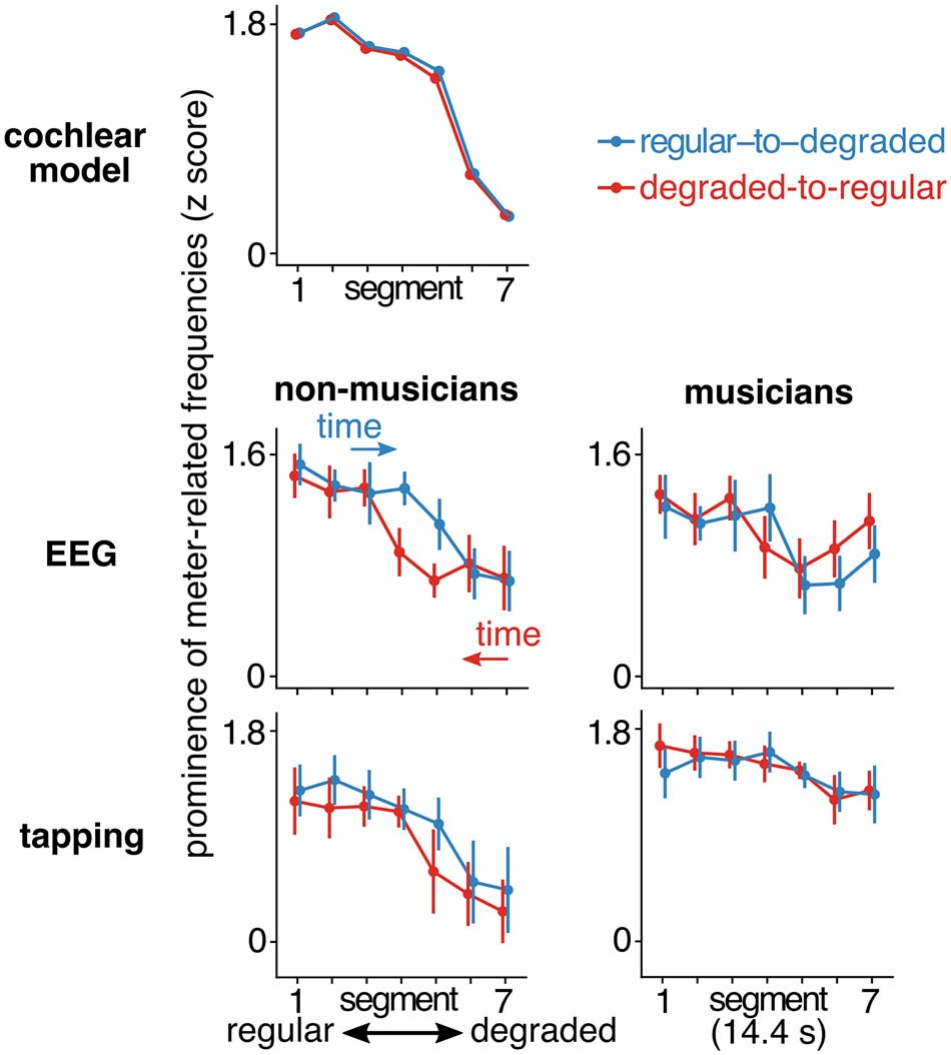
Mean *z*-scored amplitudes at meter-related frequencies in the cochlear model, EEG, and tapping response. The order of segments in the degraded-to-regular condition (red) is reversed to aid the comparison of segments with identical stimulus envelope spectra across conditions. Arrows indicate the direction of time for each condition. Mean values are shown as points, and error bars represent 95% confidence interval (Morey 2008). (Top) Cochlear model output. As intended, the prominence of meter frequencies decreased as the degradation of the sequence increased. (Middle) EEG responses plotted separately for nonmusicians (Left) and musicians (Right). Nonmusicians showed enhanced EEG responses at meter frequencies in the middle segments of the regular-to-degraded condition (blue). The EEG responses of musicians were more similar across conditions. (Bottom) Tapping responses. For nonmusicians (Left), the prominence of meter frequencies in the tapping decreased rapidly with increasing degradation. Musicians (Right) showed prominent meter frequencies in their tapping even in the degraded segments.

#### Frequency-Domain Analysis

The spectra of continuous signals from the tapping sensor exhibited prominent peaks at meter-related frequencies (Fig. 2). As depicted in Figure 3, the prominence of these frequencies in the tapping spectra evolved across segments differently for musicians and nonmusicians (*F*_6,390_ = 5.53, *P* < 0.0001, BF_10_ > 100). When comparing the two groups separately for each segment, meter frequencies were more prominent for musicians in segments 5 (β = 0.62, *t*_55.83_ = 3.39, *P* = 0.009, 95% CI = [0.25, 0.99]), 6 (β = 0.77, *t*_55.83_ = 4.2, *P* = 0.001, 95% CI = [0.4, 1.14]) and 7 (β = 0.91, *t*_55.83_ = 4.94, *P* < 0.0001, 95% CI = [0.54, 1.28]). This was due to the fact that for nonmusicians, meter frequencies significantly decreased in segments 5 (β = −0.44, *t*_396_ = −4.29, *P* = 0.001, 95% CI = [−0.65, −0.24]), 6 (β = −0.79, *t*_396_ = −7.61, *P* < 0.0001, 95% CI = [−0.99, −0.58]) and 7 (β = −0.9, *t*_396_ = −8.66, *P* < 0.0001, 95% CI = [−1.1, −0.69]) when compared with segment 1, while for musicians there was only a trend towards a decrease in segment 6 (β = −0.31, *t*_396_ = −2.97, *P* = 0.04, 95% CI = [−0.51, −0.10]). This indicates that the ability of nonmusicians to synchronize their tapping at meter frequencies deteriorated significantly once the degradation in the sensory input exceeded a critical level.

There was also a significant interaction between musical training and condition (*F*_1,390_ = 6.25, *P* = 0.01, BF_10_ = 2.4). While the overall prominence of meter frequencies was larger in the tapping of musicians for both sequence directions, this difference was more pronounced in the degraded-to-regular condition (β = 0.63, *t*_33.82_ = 3.87, *P* = 0.001, 95% CI = [0.3, 0.96]) than the regular-to-degraded condition (β = 0.43, *t*_34.11_ = 2.66, *P* = 0.02, 95% CI = [0.1, 0.76]). This was due to the fact that nonmusicians showed overall smaller prominence of meter frequencies in the degraded-to-regular condition compared with the regular-to-degraded condition (β = 0.16, *t*_396_ = 2.95, *P* = 0.01, 95% CI = [0.05, 0.27]).

#### ITI-Error Analysis

ITI-error index values further confirmed the results from the frequency domain analysis (interaction between direction and musical training, *F*_1,390_ = 10.97, *P* = 0.001, BF_10_ = 28.31), by revealing significantly less tapping error in the regular-to-degraded condition compared with the degraded-to-regular condition (β = −0.04, *t*_396_ = −4.66, *P* < 0.0001, 95% CI = [−0.05, −0.02]) for nonmusicians (Supplementary Fig. 3). Interestingly, there was no effect of segment in the analysis of ITI-error (*P*s > 0.25, BFs_10_ < 0.09). This suggests that the fast deterioration of nonmusicians’ tapping in the degraded segments, as observed in the frequency-domain analysis of tapping, was partly related to frequent changes in tapping phase. Taken together, these results suggest that nonmusicians’ tapping to the meter generally improved when the rhythm evolved from regular to degraded compared with the opposite direction, whereas musicians showed precise and stable tapping synchronization across all levels of degradation.

### Frequency-Domain Analysis of EEG

**Figure 4:**
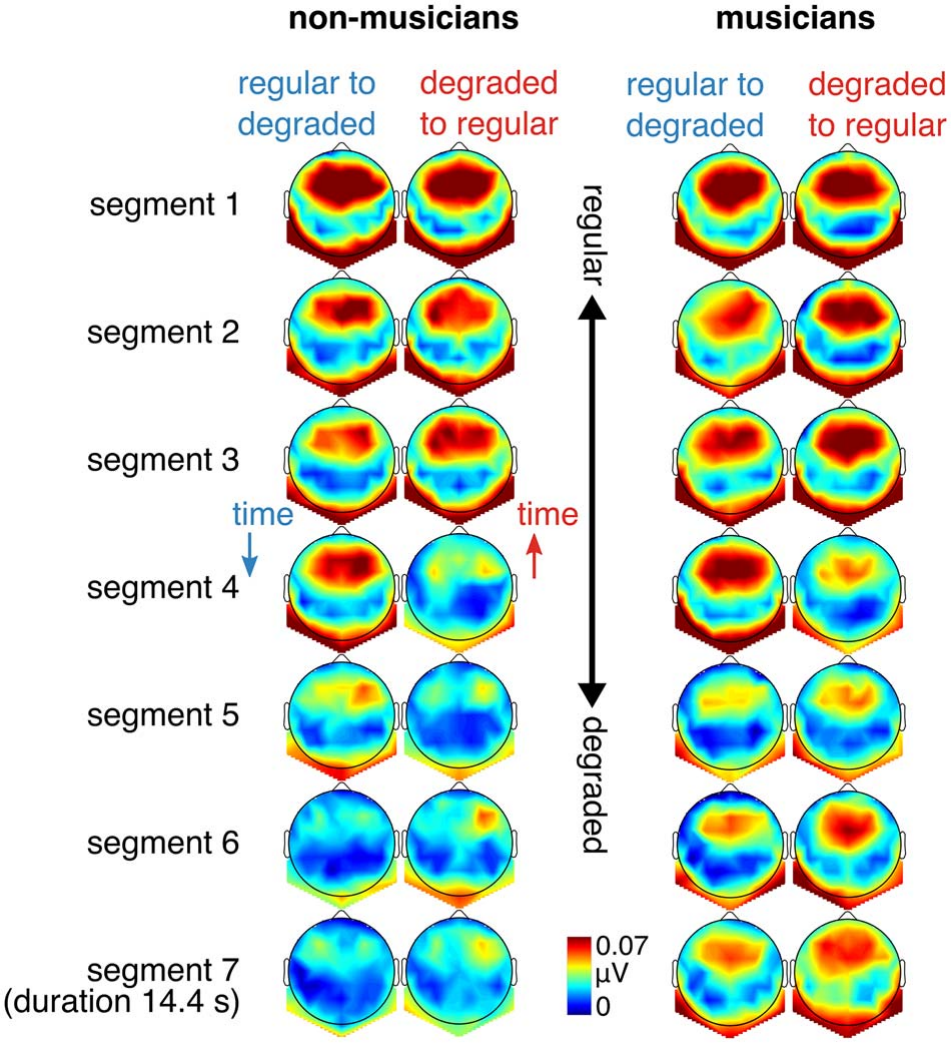
Topographies of the mean EEG amplitude at meter-related frequencies. Scalp distributions of responses across conditions and segments are shown separately for nonmusicians (Left) and musicians (Right).

EEG responses were elicited at frequencies that were expected on the basis of the auditory stimulus analysis (Fig. 2), with typical fronto-central topographies (Fig. 4), as previously observed for responses to repeating auditory rhythms (Nozaradan et al. 2012; Lenc et al. 2018).

The main aim of the current study was to examine the effect of context on the relative amplitude of EEG responses at meter-related frequencies (Fig. 3). The direction of the sequence affected the prominence of meter-related frequencies (mean *z*-scored amplitudes) in the EEG response (interaction between direction and segment, *F*_6,390_ = 4.26, *P* = 0.0004, BF_10_ = 33.70). Directly contrasting the corresponding segments between the two sequence directions revealed significantly larger meter frequencies for segment 4 (β = 0.37, *t*_396_ = 4.16, *P* = 0.0002, 95% CI = [0.20, 0.55]) in the regular-to-degraded condition compared with the opposite sequence direction. This was due to greater persistence of the response in the regular-to-degraded condition, as degradation increased. Table 1 shows the response across segments compared with the first segment, separately for musicians and nonmusicians. For nonmusicians, the response significantly decreased in segment 5, 6, and 7 in the regular-to-degraded condition. However, for the degraded-to-regular condition, there was a significant decrease already in segment 4, followed by segment 5, 6, and 7. In other words, in the segment with medium amount of degradation, the meter-related frequencies were more prominent in the EEG when regular, as opposed to degraded, input preceded this segment. Similar, although less pronounced, pattern of results was observed for musicians (decrease in segments 5 and 6 for regular-to-degraded and segments 4, 5, and 6 in the opposite direction). However, despite this apparent difference between musicians and nonmusicians, the three-way interaction between sequence direction, segment, and musical training was not significant (*F*_6,390_ = 0.71, *P* = 0.64, BF_10_ = 0.07), suggesting that context affected the neural response similarly across groups.

**Table 1:** Prominence of meter-related frequencies in the EEG response compared between the first and all subsequent segments, separately for the two sequence directions, and for musicians (*N* = 16) and nonmusicians (*N* = 16)

Musical training	Direction	Contrast segments	Estimate	df	*t*	Lower CI	Upper CI	*P*-value
Nonmusicians	Regular-to-degraded	2–1	−0.15	390	−1.19	−0.49	0.19	1.00
		3–1	−0.21	390	−1.63	−0.54	0.13	1.00
		4–1	−0.17	390	−1.36	−0.51	0.16	1.00
		5–1	−0.43	390	−3.40	−0.77	−0.09	0.02^*^
		6–1	−0.79	390	−6.21	−1.12	−0.45	<0.0001^*^
		7–1	−0.84	390	−6.62	−1.18	−0.50	<0.0001^*^
	Degraded-to-regular	2–1	−0.12	390	−0.91	−0.45	0.22	1.00
		3–1	−0.09	390	−0.69	−0.42	0.25	1.00
		4–1	−0.55	390	−4.33	−0.89	−0.21	0.0004^*^
		5–1	−0.76	390	−5.95	−1.09	−0.42	<0.0001^*^
		6–1	−0.63	390	−4.98	−0.97	−0.30	<0.0001^*^
		7–1	−0.74	390	−5.81	−1.07	−0.40	<0.0001^*^
Musicians	Regular-to-degraded	2–1	−0.12	390	−0.94	−0.46	0.22	1.00
		3–1	−0.06	390	−0.50	−0.40	0.27	1.00
		4–1	−0.01	390	−0.06	−0.34	0.33	1.00
		5–1	−0.57	390	−4.45	−0.90	−0.23	0.0003^*^
		6–1	−0.55	390	−4.35	−0.89	−0.22	0.0004^*^
		7–1	−0.34	390	−2.68	−0.68	0.00	0.18
	Degraded-to-regular	2–1	−0.18	390	−1.41	−0.52	0.16	1.00
		3–1	−0.03	390	−0.21	−0.36	0.31	1.00
		4–1	−0.38	390	−3.01	−0.72	−0.05	0.07
		5–1	−0.53	390	−4.21	−0.87	−0.20	0.0008^*^
		6–1	−0.39	390	−3.09	−0.73	−0.06	0.05
		7–1	−0.19	390	−1.52	−0.53	0.14	1.00

Note: CIs represent 95% confidence intervals.

^*^ *P* < 0.05.

Furthermore, there was an interaction between musical training and segment (*F*_6,390_ = 4.35, *P* = 0.0003, BF_10_ = 41.70). However, this effect seemed primarily driven by greater selective response at meter-related frequencies in segment 7 for musicians, which did not reach significance in the posthoc contrasts (β = 0.30, *t*_109.06_ = 2.57, *P* = 0.08, 95% CI = [0.07, 0.54]). Finally, musical training interacted with sequence direction (*F*_1,390_ = 9.03, *P* = 0.003, BF_10_ = 6.51). However, posthoc contrasts did not reveal significant differences between musicians and nonmusicians in either condition (*P*s > 0.13).

A number of control analyses were done to confirm that the sequence direction effects observed here were not spurious (see Supplementary Results). Specifically, these control analyses showed that the context effect (1) could not be explained by head movement artifact or (2) low-level nonlinear auditory processing of the inputs, and (3) was not a spurious effect of the standardization, or (4) noise subtraction procedure applied to the EEGdata.

## Discussion

Our results show direct evidence for sensitivity to recent auditory context in neural responses to rhythmic inputs. In the EEG, we observed a selective enhancement of meter-related frequencies that persisted when the acoustic cues guiding meter perception were gradually degraded in the stimulus. Conversely, these meter-related frequencies were less prominent in the neural response when the preceding input lacked acoustic cues to guide meter perception. Moreover, this context effect seemed stronger in participants with no formal musical training, who (1) demonstrated sensitivity to context in their ability to tap along with the meter, and (2) whose tapping deteriorated when it was not supported by acoustic cues in the stimulus. In contrast, the context effect appeared weaker in musicians, whose ability to maintain a meter was robust to stimulus degradation, and independent of context, as observed in the tapping session. Together, these results demonstrate that perceptual organization of a rhythmic stimulus is not solely determined by low-level features of the sensory input but also involves integration of prior experience, as reflected in the elicited neural activity.

Importantly, our stimulus design ensured that low-level input properties such as envelope spectra could not fully account for the observed neural responses. Moreover, the context effect observed here was unlikely to be explained by nonlinearities at the early stages of the auditory pathway (as indicated by the analysis of our stimuli with a biologically plausible model of the auditory periphery), or the overall gain (as we used a relative measure of response prominence). Instead, the context effect could be explained by selective neural enhancement of meter-related frequencies as a function of prior prominence of these frequencies in the sensory input.

### No One-to-One Mapping Between Sensory Input and Perception

#### Robust Perception

Human perception shows remarkable robustness to degraded sensory input across domains (Shannon et al. 1995; Schwiedrzik et al. 2018). For instance, while under certain conditions the perception of a visual object or a speech utterance can be largely determined by the physical features of the sensory input, in real-world noisy situations the mapping between the input and perceptual experience is far from trivial. Our results show that similar processes may be at work in perceptual organization of rhythm, especially for individuals with musical training. We found that musicians were able to precisely synchronize their tapping to the perceived meter even when this meter could not be clearly determined from the stimulus features alone. This is in line with previous evidence that musical training generally leads to superior precision of meter representation (Rüsseler et al. 2002; Brochard et al. 2003; Geiser et al. 2010; Lappe et al. 2011), with a high degree of invariance with respect to the rhythmic stimulus (Repp 2007, 2010; Repp et al. 2008; Su and Pöppel 2012).

#### Sensitivity to Context

Further evidence against a one-to-one mapping between acoustic input and perceptual output is provided by the effect of recent context we observed in the tapping and in the EEG response. These results suggest that perception of meter in degraded rhythmic input can be facilitated when the directly preceding input provides clear sensory cues to the meter periodicities. While effects of recent context have been investigated in single-interval timing (Drake and Botte 1993; Large 2000; McAuley and Jones 2003; Jazayeri and Shadlen 2010; Cicchini et al. 2012) and rhythmic pattern perception (Desain and Honing 2003), they remain under-explored with respect to perceptual organization of rhythmic patterns (Cameron and Grahn 2016). The current results thus constitute a step forward in our understanding of how the brain dynamically builds representation of complex patterns of time intervals.

The fact that these context effects were stronger in participants with no musical training is consistent with the hypothesis that influence of prior context increases as the uncertainty of the current representation increases (Cicchini et al. 2018; Cicchini and Burr 2018). Nonmusicians, whose meter perception was overall less robust to input degradation, would rely more on the recent context to make better sense of the degraded input (see Cicchini et al. 2012 for similar findings in single time interval reproduction). The context effect observed here is also similar to widely studied phenomena in visual object recognition and language domains, where perception of objects from impoverished inputs can be enhanced by prior exposure to the intact version of the stimulus (Bruner and Potter 1964; Dolan et al. 1997; Kleinschmidt et al. 2002; Ahissar and Hochstein 2004; Melloni et al. 2011; Teufel et al. 2015), or even through higher-level semantic context (Eger et al. 2007; Hervais-Adelman et al. 2008; Esterman and Yantis 2010; Sohoglu et al. 2014; Stein and Peelen 2015). Both types of perceptual enhancements have been linked to neural responses across a widespread network, involving sensory and frontal cortices (Kleinschmidt et al. 2002; Hegdé and Kersten 2010; Sohoglu et al. 2012; Sohoglu and Davis 2016). Moreover, there is evidence suggesting that the underlying mechanism might involve top–down modulations biasing processing of input features in sensory areas towards greater similarity with the expected category (Hsieh et al. 2010; Holdgraf et al. 2016; Leonard et al. 2016; St. John-Saaltink et al. 2016). While our method does not address the neural network mediating the context effects observed here, our results provide a new critical piece of knowledge on the integration of sensory input with context. That is, brain activity elicited at behaviorally relevant frequencies is significantly modulated by the prominence of these frequencies in recent input. These findings may thus provide a basis to further investigations of the nature of neural representations of rhythmic input, using a similar design combined with a range of neuroimaging methods including intracerebral EEG (Grahn and Rowe 2013; Chemin et al. 2014; Rajendran et al. 2017; Mendoza et al. 2018; Narain et al. 2018; Gámez et al. 2019; Sohn et al. 2019).

### Evidence Against Evoked Responses Passively Tracking Low-Level Acoustic Features of the Rhythmic Input

Increasing evidence converges towards the view that during meter perception, the brain transforms the sensory input (a sequence of events in time) towards the metrical category (a nested set of periodic pulses), and this transformation can be observed as a selective increase of brain response at meter-related frequencies (Nozaradan et al. 2012, 2018; Nozaradan, Mouraux, et al. 2016a; Nozaradan, Keller, et al. 2017a). Importantly, this transformation is not fixed or mechanistic, but can be flexibly shaped by the spectral acoustic context (Lenc et al. 2018), prior body movement (Chemin et al. 2014), or behavioral goals (Nozaradan et al. 2011). Here, we add to this evidence by showing that this transformation can be dynamically shaped by preceding input and even without overt movement. Together, these results thus provide strong evidence against the view that this selective increase of brain response at meter-related frequencies reflects passive tracking of low-level features of the rhythm (Large et al. 2015; Daube et al. 2019; Rimmele et al. 2020). Instead, the data suggest that this measure is (1) behaviorally relevant, and (2) reflects transformation from acoustic features towards higher-level categories, in line with recent work on speech (Ding and Simon 2012; Mesgarani and Chang 2012; Di Liberto et al. 2015, 2019; Brodbeck et al. 2018; Broderick et al. 2018) and melody perception (Di Liberto et al. 2020; Sankaran et al. 2020).

Moreover, the approach used in the current study goes beyond the common assumption that better alignment of neural response with stimulus envelope necessarily reflects better processing (Park et al. 2015; Etard and Reichenbach 2019; Harding et al. 2019; Herff et al. 2019; Fiveash et al. 2020; Wollman et al. 2020). Specifically, instead of looking for precise reconstruction of low-level features such as envelope periodicity using, for example, input–output coherence or regression analysis, the current study aimed to investigate dynamic processes that continuously transform sensory input towards invariant perceptual categories (Ley et al. 2014; Kuchibhotla and Bathellier 2018; Broderick et al. 2019; Yi et al. 2019). The input–output mapping approach used here allowed us to uncover these processes while ensuring that the results are not driven by (1) acoustic confounds, (2) overall gain of the response, or (3) low-level nonlinear auditory processes.

### Context Effect is Short-Lived in Neural Activity but Long-Lasting in Behavior

In the current study, the contextual enhancement of meter-related frequencies in the EEG was relatively short-lived, that is, lasting around one 14-s long segment. These observations demonstrate that the influence of prior acoustic context on EEG responses might have a short time constant, only affecting the processing of directly following rhythmic material. Such short-lived integrative mechanism would thus make the system both robust to momentary changes in the sensory input (e.g., syncopation, Sioros et al. 2014) and flexible enough to adjust meter perception under persisting counterevidence from the sensory input (London 2004; Fitch and Rosenfeld 2007).

The short time constant observed here could also be due to the stimulus sequence design combined with a context effect restricted to inputs up to a certain level of input degradation. Indeed, while perception across domains is remarkably robust to sensory degradation, the perceptual system is limited in terms of the minimal amount of sensory cues required to elicit a percept (for evidence of these limits in meter perception see e.g., Nozaradan et al. 2012; Witek et al. 2014; Vuust et al. 2018; Matthews et al. 2020). Even though prior context may significantly shift this limit, perceptual organization may be lost once the cues in the sensory input are too degraded. Consequently, the effects of prior context would be confined to inputs with medium amounts of degradation, thus explaining why we did not observe selective enhancement of meter frequencies in response to the most degraded sections of the sequences.

In contrast to the neural response, the effect of recent context in sensory-motor synchronization was spread across all segments. This difference between neural response and sensory-motor synchronization is in line with recent studies showing that synchronized movement can directly (Nozaradan et al. 2013; Nozaradan, Schönwiesner, et al. 2016c; Morillon and Baillet 2017; Yon et al. 2018) and prospectively (Lahav et al. 2007; Chemin et al. 2014) affect sound processing in the brain. While it has been previously shown that overt movement can facilitate extraction of a periodic pulse from complex rhythmic sequences (Su and Pöppel 2012), our results suggest that in certain situations, overt movement may impede extraction of a periodic meter. This could be specific to situations similar to the current study, where the preceding movement is desynchronized, possibly preventing extraction of regularities gradually emerging in the sensory input. Alternatively, it could be that the location of the prior-context benefit within the sequences was variable across trials, yielding generally improved performance in the regular-to-degraded sequence after averaging. These possibilities remain to be investigated with larger samples allowing for more detailed tapping analyses.

## Conclusion

Together, our results demonstrate that, similar to high-level perceptual organization in other domains, meter can emerge from highly complex and degraded sensory inputs. At the same time, the robustness to input degradation is limited (Witek et al. 2014; Vuust et al. 2018) and these limits depend on context and prior experience. These observations highlight the predictive nature of perceptual processing and the importance of endogenous information (such as prior knowledge and expectations) in shaping the processing of sensory signals across domains (de Lange et al. 2018; Demarchi et al. 2019; Koelsch et al. 2019).

A common assumption in the neuroscientific literature is that meter perception can be predicted from the acoustic features of the rhythmic stimulus. In other words, rhythms with a good fit between the distribution of acoustic events and hypothetical pulses comprising meter (i.e., regular rhythms) are assumed to induce “strong” meter perception, whereas degraded rhythms are expected to induce “weak” or no meter perception (Povel and Essens 1985; Grahn and Brett 2007; Bengtsson et al. 2009; Grube and Griffiths 2009; Kung et al. 2013). Together, our findings caution against a too strict stimulus-centered view, suggesting that prior experience at short and long timescales is critical to understand the mapping between sensory input and perception of rhythm.

## Supplementary Material

XPSyncSweep_Supplement_CerebCortex_TL_tgaa037
