# Supplementary material for: Neural and Behavioral Evidence for Frequency-Selective Context Effects in Rhythm Processing in Humans
Source: Cereb Cortex Commun. 2020 Jul 28;1(1):tgaa037. doi: 10.1093/texcom/tgaa037 (PMC8152888; doi:10.1093/texcom/tgaa037)
Supplement: XPSyncSweep_Supplement_CerebCortex_TL_tgaa037 [file xpsyncsweep_supplement_cerebcortex_tl_tgaa037.pdf]

Supplementary Material for

Neural and behavioral evidence for frequency-selective context effects in rhythm processing in humans

Tomas Lenc*, Peter E. Keller, Manuel Varlet, Sylvie Nozaradan

*To whom correspondence should be addressed

Email: T.Lenc@westernsydney.edu.au

This PDF file includes:

Supplementary Results

Figures S1-3

Supplementary References

Supplementary Results: Control analyses of EEG data

Contribution of low-level nonlinear auditory processes. Direct comparison between the cochlear model and EEG data indicated that the results are unlikely to be fully explained by nonlinearities in the early stages of the auditory pathway. The difference in prominence of meter-related frequencies between EEG and cochlear model significantly depended on the direction of the sequence (interaction between Direction and Segment, $F_{6,838} = 8.42$, $P < 0.0001$, $BF_{10} > 100$) for segment 4 ($\beta = 0.36$, $t_{844} = 5.91$, $P < 0.0001$, 95% CI = [0.24, 0.48]). Similar to the main analysis, there was an interaction of musical training and segment ($F_{6,838} = 9.34$, $P < 0.0001$, $BF_{10} > 100$), driven by greater response at meter frequencies for musicians in segment 7 ($\beta = 0.30$, $t_{63.22} = 2.99$, $P = 0.03$, 95% CI = [0.10, 0.51]). Additionally, there was an interaction between musical training and sequence direction ($F_{1,838} = 19.4$, $P < 0.0001$, $BF_{10} > 100$). Again, directly contrasting musicians and non-musicians for each condition did not yield significant differences (Ps > 0.12).

Together, these results indicate that even after accounting for the response variability explained by the cochlear model, EEG responses at meter-related frequencies were significantly affected by sequence direction.

Raw amplitudes. Z-scoring EEG amplitude values across a set of frequencies was used to assess the selective variations in the amplitudes at meter-related frequencies in a manner that minimized the contribution of the overall gain. However, to demonstrate that this standardization procedure was not responsible for the context effect observed in the current study, we carried out a control analysis of the EEG response without any normalization, i.e. using raw amplitude values from the EEG spectra averaged over meter-related frequencies as the dependent measure. The amplitude at meter-related frequencies was affected by sequence direction (interaction between Direction and Segment, $F_{6,390} = 6.97$, $P < 0001$, $BF_{10} > 100$), and this was due to significantly larger amplitude at meter frequencies in the regular-to-degraded condition for segment 4 ($\beta = 0.37$, $t_{396} = 4.16$, $P = 0.0003$, 95% CI = [0.20, 0.55]). There was also a significant interaction between musical training and segment ($F_{6,390} = 4.44$, $P = 0.0002$, $BF_{10} = 39.56$), due to the marginally higher

prominence of meter frequencies in segment 7 for musicians (β = 0.30, $t_{109.06}$ = 2.57, P = 0.08, 95% CI = [0.07, 0.54]). These results suggest that z-scoring EEG amplitudes alone is not likely to explain the current results.

Without noise subtraction. The prominence of meter-related frequencies extracted from EEG spectra without noise subtraction was affected by sequence direction (interaction between Direction and Segment, $F_{6,390}$ = 5.30, P < 0.0001, BF_{10} > 100), due to greater meter z score in segment 4 (β = 0.22, t_{396} = 3.30, P = 0.007, 95% CI = [0.09, 0.35]). Interaction between musical training and segment also reached significance ($F_{1,390}$ = 2.72, P = 0.01, BF_{10} = 1.45), but there was no significant difference for either segment separately (Ps > 0.47). These results suggest that noise subtraction alone is not likely to explain the current results.

Head movement analysis. The prominence of meter-related frequencies in the head movement data was not significantly affected by sequence direction (Ps > 0.44, BFs_{10} < 0.32). There was a weak but significant main effect of Segment ($F_{6,390}$ = 2.32, P = 0.03, BF_{10} = 0.35). A significant linear trend (β = -1.04, t_{409} = -3.26, P = 0.001, 95% CI = [-1.67, -0.41]) suggested that participants had a tendency to synchronize subtle head movements with the meter when the stimulus was more regular. Together, this control analysis suggests that the observed EEG effects are unlikely to be explained by head movement artifacts.

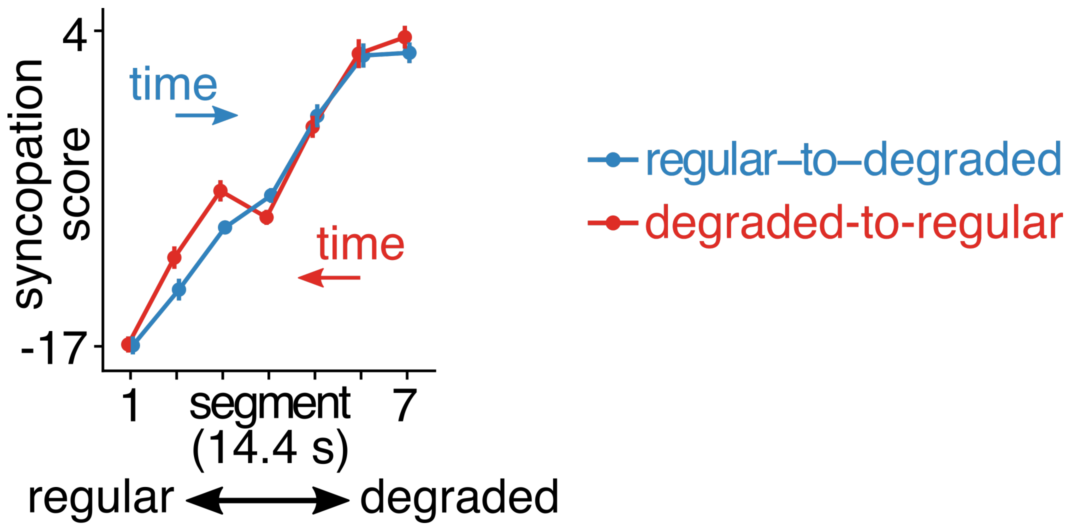

Figure S1. Analysis of the stimulus sequences used in the EEG session. Syncopation scores are averaged across the 15 trials separately for each condition, with arrows indicating the direction of time for each condition. Error bars represent 95% confidence interval (Morey 2008).

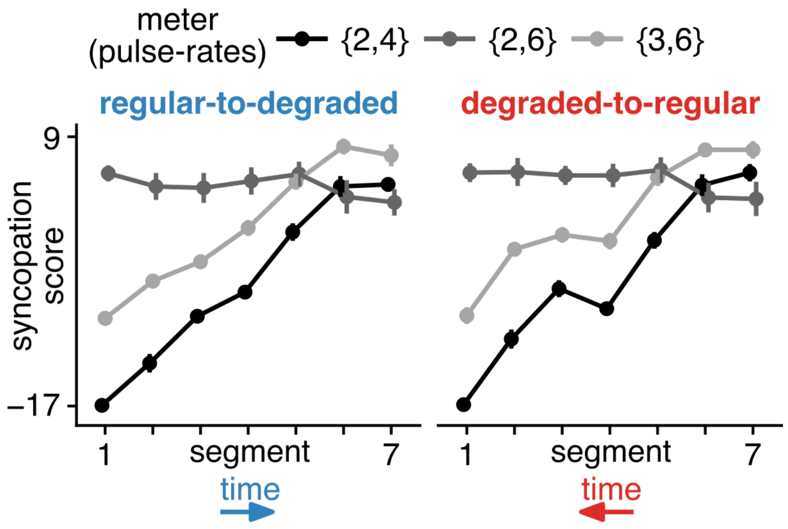

Figure S2. Evolution of syncopation scores across segments, assuming different metrical interpretations of the sequences (nested pulses at rates of {2,4}, {3,6}, and {2,6} events). Syncopation scores for {2,6}-meter remained high throughout the sequence, whereas syncopation scores for {3,6}-meter increased monotonically along with the {2,4}-meter used in the main analysis. This suggests that the sequences did not change between different meters, but gradually changed from providing clear cues to a {2,4} meter to containing little cues to any regular meter.

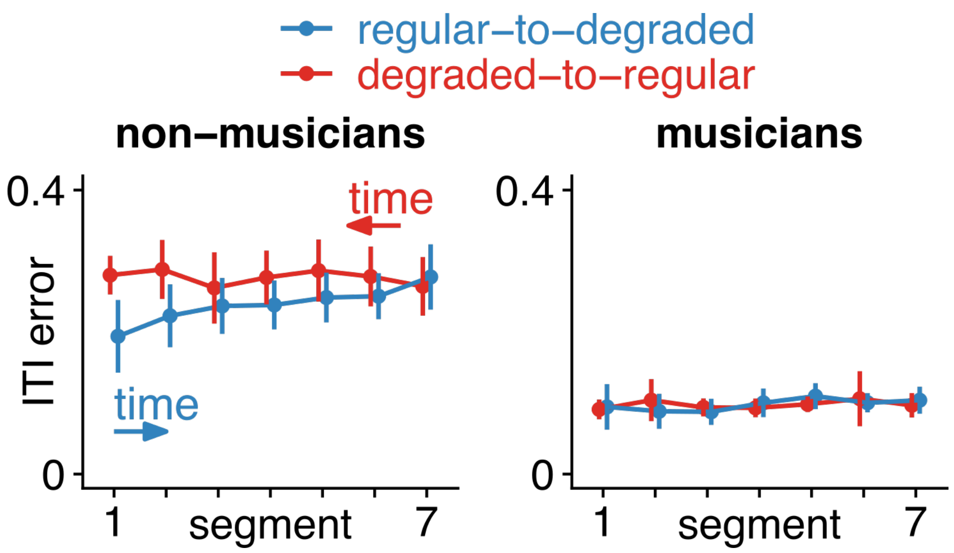

Figure S3. Analysis of inter-tap interval (ITI) error across sequence directions and segments. The order of segments in the degraded-to-regular condition (red) is reversed in time (indicated by arrows) to aid the comparison of segments with equivalent amount of degradation. Mean values are shown as points, and error bars represent 95% confidence interval (Morey 2008). (Left) Non-musicians performed worse (larger error between their ITIs and meter periodicities) in the degraded-to-regular condition. (Right) Musicians' ITI error was low and stable over segments, suggesting that they produced ITIs close to the meter periodicities irrespective of stimulus degradation and context.

Supplementary References

Morey RD. 2008. Confidence Intervals from Normalized Data: A correction to Cousineau (2005). Tutor Quant Methods Psychol. 4:61–64.
